# Revealing the Impact of Pasteurization and Derivatization Chemistry on the Fatty Acid Profile of Dairy Cream: A Comparative Approach

**DOI:** 10.3390/foods14223815

**Published:** 2025-11-07

**Authors:** Aleksandra Bogumiła Florkiewicz, Gaja Gużewska, Izabela Arendowska, Agnieszka Ludwiczak, Joanna Rudnicka, Małgorzata Szultka-Młyńska, Tomasz Ligor, Paweł Piotr Pomastowski

**Affiliations:** 1 Centre for Modern Interdisciplinary Technologies, Nicolaus Copernicus University in Toruń, 87-100 Toruń, Polandjrud@umk.pl (J.R.); mszultka@umk.pl (M.S.-M.); p.pomastowski@umk.pl (P.P.P.); 2 Department of Environmental Chemistry and Bioanalytics, Faculty of Chemistry, Nicolaus Copernicus University in Toruń, 87-100 Toruń, Poland; 3 Polmlek Grudziądz Ltd., 86-300 Grudziądz, Poland; 4 Department of Histology and Embryology, Institute of Medical Sciences, College of Medical Sciences, University of Rzeszów, 35-025 Rzeszów, Poland; 5 Department of Immunology, Institute of Biology, Faculty of Biological and Veterinary Sciences, Nicolaus Copernicus University in Toruń, 87-100 Toruń, Poland

**Keywords:** cream, dairy products, derivatizations, fatty acids, fatty acid methyl esters, gas chromatography

## Abstract

Milk and dairy products are a vital source of nutrients. This study aimed to evaluate the impact of pasteurization and the choice of derivatization method on the fatty acid (FA) profile in cream, a milk fat-rich product. Sixty cream samples (pre- and post-pasteurization) were analyzed. Two derivatization procedures were used: acid-catalyzed (1% H_2_SO_4_ in methanol following hexane extraction) and alkali-catalyzed (0.2 M KOH in methanol). FA methyl esters (FAMEs) were quantified using GC–FID. A total of 34 FAs were detected. The acid derivatization method was significantly more efficient for quantification, yielding higher overall FA concentrations (e.g., 302.26 μg/mL vs. 62.66 μg/mL pre-pasteurization). Pasteurization significantly altered the FA profile by reducing the overall content of FAs (especially SFAs and PUFAs), suggesting thermal degradation. Conversely, concentrations of FAs with unusual chain lengths (e.g., C15:1, C17:0) increased, likely due to release from complex lipids. The FA profile in cream is sensitive to processing. Acid-catalyzed derivatization is the recommended method for accurate quantitative FA analysis in cream. The stability of milk fat confirms its importance for product quality and potential use in various bioformulations.

## 1. Introduction

The production process in modern dairies begins with specialized departments responsible for receiving milk from farmers, accurately quantifying and registering its volume within the control system. Dairy cream is a key ingredient used in the manufacturing of various products, including butter, ice cream, and sour cream [1]. Its formation involves a centrifugation process that separates the fat fraction from the raw milk. Subsequently, skim milk is added to achieve the desired, standardized fat content.

Due to its properties, a pH of approximately 6.7 and high-water activity (around 0.97), cream is a highly perishable product, thus requiring preservation to extend its shelf life. The standard technique for most retail and industrial cream is thermal pasteurization. To prevent phase separation (layering) and ensure microbiological purity, cream is also homogenized and pasteurized. Pasteurization is critical because its primary goal is the elimination of vegetative microorganisms (both pathogenic and spoilage-causing) and the inactivation of enzymes, which effectively prolongs the product’s durability. The manufacturing of sour cream (cultured cream) involves the same initial processing steps, but the product is additionally chilled and inoculated with lactic acid bacteria (LAB) [2,3,4].

Milk fats are notable for their extraordinary diversity of fatty acids (FA), which include compounds such as conjugated linoleic acid (CLA), omega-3 (EPA, DHA), and omega-6 (LA, AA, GLA). These compounds fulfill a variety of biological functions, ranging from providing energy to supporting cellular and metabolic functions. It is also important to note that some of these fatty acids are essential in the human diet due to their anti-inflammatory properties and beneficial effects on the cardiovascular system [5,6].

The choice of analysis method primarily depends on the properties of the measured components. The most commonly used instrumental methods in food analysis include UV-Vis spectrophotometry, near-infrared spectroscopy (NIRS), NMR spectroscopy, and gas chromatography (GC), liquid chromatography (LC), and hyphenated techniques such as gas chromatography-mass spectrometry (GC–MS) and liquid chromatography–mass spectrometry (LC–MS) [7,8]. Various derivatization methods are required to prepare UV-absorbing derivatives because of the lack of suitable chromophores in underivatized FAs. However, derivatization increases analysis time and complicates the method. Therefore, LC–UV analysis is rarely used to detect FAs. When dealing with complex matrices such as sour cream, specific challenges arise. Long-chain fatty acids (FAs) exhibit low volatility and strong polarity, and at elevated temperatures they may undergo polymerization, decarboxylation, cracking, or other undesirable reactions. Due to these properties and thermal instability, samples require special preparation prior to GC analysis, often using derivatization, which increases volatility and improves analytical quality. Furthermore, technological processes such as pasteurization can affect lipid stability and increase the content of free fatty acids (FFAs), which poses a challenge for precise measurement of the entire FA pool [9]. The presence of carboxyl groups affects the sensitivity of the analysis and can lead to peak tailing on chromatograms, which poses a significant problem in result interpretation. The derivation process is used to increase the effectiveness of the analysis. This procedure transforms FAs into their derivatives, which are more volatile, thermally stable, and less polar than the original substances, thus significantly improving the quality of chromatographic analyses [10]. The most commonly used detector for FA detection is the flame ionization detector (FID), known for its high accuracy, sensitivity, stability, short response time, and linearity over a wide concentration range [11,12]. GC is also frequently combined with the MS for highly selective and sensitive quantitative analysis as well as high resolution [13]. Different types of LC (reversed and normal phase) are also used to analyze and separate FAs [14]. In this technique, FAs are converted into different derivatives. Derivatization can overcome limitations, such as tailing peaks and low detector sensitivity, because of the formation of a few polar compounds, which LC can quickly analyze. In the context of dairy, research progress to date has confirmed the significant impact of pasteurization on the physicochemical properties of lipids, including the potential release of FFAs, which directly impacts the required derivatization method [7]. FAME production depends on the type of FAs—acidic reagents are used for free fatty acids (FFAs), and alkaline methanolysis is applied for acylglycerols [15]. Fatty acids in the form of FAMEs can be analyzed using capillary columns of varying polarities. Analyses are carried out on capillary columns, and for FA mixtures—at programmed temperatures [16]. This allows for the complete separation of all mixture components, significantly facilitating their identification and quantitative assessment. In contrast to conventional analytical techniques, NIRS requires reference values and corresponding chemometric techniques during calibration and allows rapid measurement of several traits in a single analysis without chemical reagents [17].

Therefore, developing new methodological approaches for the analysis of active ingredients in food samples, including dairy products, is crucial to improving the precision and efficiency of chemical or bioanalytical analyses in consumer products. Despite the understanding of the chemical foundations, comprehensive, direct comparisons of the efficiency of acid- and alkaline-catalyzed derivatization, which also take into account real-world dairy processing conditions, are lacking.

This study addresses this gap and provides a novel approach by systematically comparing analytical methods for determining FAs in cream samples, with particular emphasis on the effect of pasteurization and comparing the efficiency of derivatization in acidic and alkaline environments. Target compounds were identified using GC-FID.

## 2. Materials and Methods

### 2.1. Cream Material

Cream samples were provided by a local dairy Polmlek Grudziądz Ltd. (Grudziądz, Poland). Cream was collected and delivered fresh in two batches (before and after pasteurization) every two weeks for about three months (February–April), giving a total of 60 samples of cream. It was collected in sterile plastic containers, stored at 4 °C from the moment of collection until transport to the Centre for Modern Interdisciplinary Technologies of Nicolaus Copernicus University in Toruń. Cream samples were allowed to reach room temperature (20 ± 1 °C) and mixed before aliquot collection. Physicochemical parameters of 30 cream samples before and of 30 cream samples after pasteurization are presented in Supplementary Table S1, including parameters such as acidity in Soxhlet–Henkel degrees (°SH), pH values, as well as fat, protein, lactose and dry matter content expressed in %.

### 2.2. Extraction, Purification and Identification of Selected Fatty Acids

High-purity, GC-MS-grade chemicals were used in the study, including water, acetonitrile, acetone, chloroform, sodium chloride, sulfuric acid (VI), hexane, methanol, potassium carbonate from Sigma-Aldrich (Steinheim, Germany). Small laboratory equipment and plastics were purchased from Alchem (Toruń, Poland). Deionized water (H_2_O) was obtained using a system from MerckMillipore Water Purification Systems (Schaffhausen, Switzerland).

For quantitative analyses and master curves were prepared, the following solutions of 37 FAs mixture (Supelco 37 Component FAME Mix; Sigma-Aldrich, Schnelldorf, Germany) were also made, i.e., stock (1 mL FAME solution was dissolved in 1 mL methanol) and standard (FAME solutions of 0.01–10 ng/mL) were prepared by diluting an appropriate volume of the stock solution. Methanol was used for dilutions. Concentrations are expressed in μg FAME per ml of original (collected) cream sample.

#### 2.2.1. Derivatization in Acid Medium

The endogenous level of FA in cream samples was measured using GC–FID. The FA methylation procedure was based on methods described by Christie [18] and Martínez et al. [19]. Carefully, 1 mL of previously mixed cream was taken into 15 mL Falcon tubes, and FA extraction was performed using 1 mL of hexane. The mixture was vortexed, centrifuged (4000× *g* for 10 min), and left for 2 min to allow phase separation. This process was repeated twice. The combined organic phase fractions were dried under a nitrogen stream. Methylation was carried out with 2 mL of a 1% sulfuric acid solution in methanol, and the tube was placed in a water bath (60 °C, 3 h) (Julabo, Święcice k/Błonia, Poland). After cooling, 2 mL of saturated 6% sodium chloride and 1.5 mL of hexane were added to the mixture, centrifuged as described earlier, and the supernatant was retained. The extraction was repeated twice. The combined hexane extracts were treated with 4% potassium carbonate, transferred to new tubes, and dried under a nitrogen stream. After drying, the fractions were dissolved in 1 mL of methanol and analyzed with the use of GC–FID.

#### 2.2.2. Derivatization in Alkaline Medium

The procedure for alkali-catalyzed fatty acid methanolysis was based on the methods described by Christie [18] and modified accordingly. Circa 0.5 mL of previously mixed cream was taken into a 15 mL Falcon tube, and the samples were then extracted with 2 mL of a chloroform and methanol mixture in a 2:1 ratio (*v*/*v*). The organic phase was transferred to the clean 15 mL Falcon tubes and evaporated to dryness under a stream of nitrogen. To the prepared samples, 3 mL of a derivatization reagent, consisting of a 0.2 M potassium hydroxide solution dissolved in methanol, was added, and the samples were incubated for 1 h in a water bath (Julabo, Święcice k/Błonia, Poland) at 75 °C. After this time and after briefly cooling the samples, 1 mL of water was added, and the resulting FAME was extracted with 1 mL of hexane. The extracts were transferred to new 15 mL Falcon tubes and dried using 2 mL of 5% potassium carbonate. The organic phase was collected into new 15 mL Falcon tubes and evaporated to dryness under a stream of nitrogen. The precipitates were dissolved in 1 mL of methanol and analyzed with the use of GC–FID.

### 2.3. Conditions for Chromatographic Analysis

Measurements were conducted using a 7820A gas chromatograph with a flame ionization detector (GC–FID) (Agilent Technologies, Santa Clara, CA, USA). The chromatograph was equipped with an autosampler G4513A (Agilent Technologies, Santa Clara, CA, USA) and fitted with an ZB-WAX column with the dimensions 30 m × 0.25 mm × 0.25 μm (Agilent Technologies, Santa Clara, CA, USA). Aliquot of each sample (2 μL) was automatically injected using a split/splitless injector with a split ratio of 1:15. Helium served as the carrier gas at a flow rate of 1.9 mL/min. The injector temperature was set to 240 °C, the temperature program of the chromatographic oven was as follows: 60 °C (2 min), ramping at 13 °C/min to 150 °C (0 min), followed by a ramp of 2 °C/min to 230 °C (6 min). Helium with a purity of 99.999% was used as the carrier gas. The carrier gas flow rate was 35 mL/min.

### 2.4. Statistical Analysis

All experiments were conducted in three replicates. The content of the analyzed compounds was determined based on the relationship between peak area and analyte concentration. A calibration curve was created using eight calibration standards. The equations and coefficients of determination (R^2^) were calculated using the least squares regression method. The acceptance criterion was set at R^2^ > 0.998 (Supplementary Table S2). The statistical assessment of the calibration curves was performed by calculating the standard deviations (SD). The statistical analyses and visualizations were conducted using the Python programming environment (version 3.9) and Python data analysis library (the pandas library—version 2.2.3) for data analysis and Matplotlib (version 3.7.4) for graphical representation.

**Figure 1:**
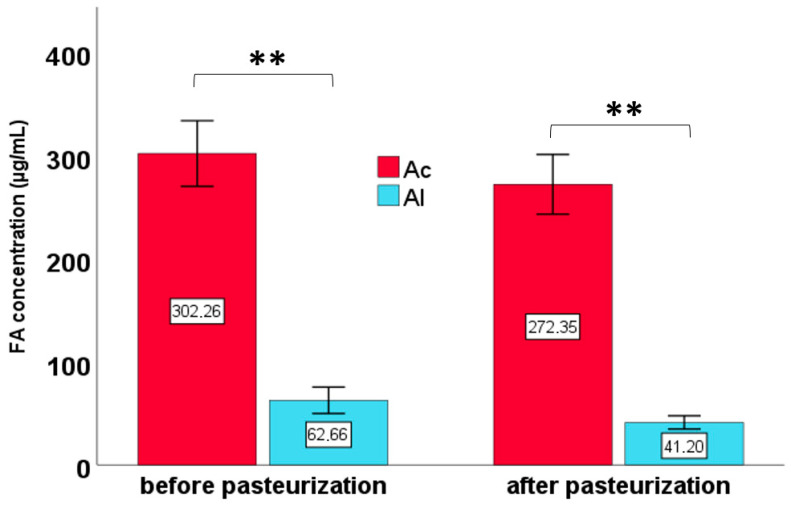
Fatty acid (FA) concentrations across pasteurization phases and derivatization types. Asterisks indicate statistically significant differences at *p* ≤ 0.01. Error bars represent 95% confidence intervals. Abbreviations: Ac—acid derivatization; Al—alkaline derivatization.

**Figure 2:**
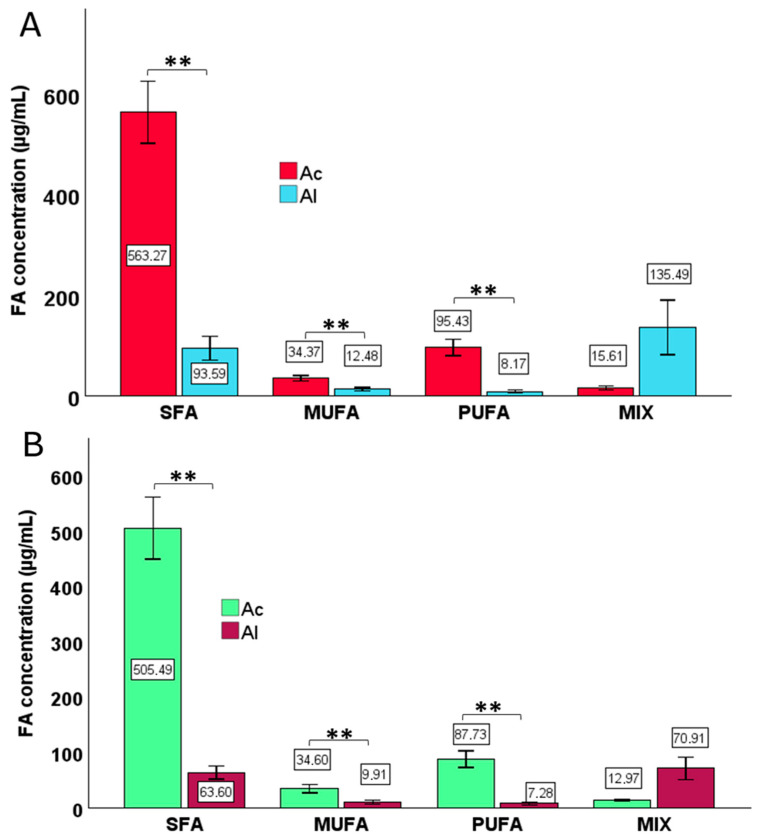
Changes in fatty acid (FA) concentrations within specific lipid classes (SFA, MUFA, PUFA, MIX) depending on the type of derivatization for cream samples collected: (**A**) before pasteurization, and (**B**) after pasteurization. SFA—saturated fatty acids, MUFA—monounsaturated fatty acids, and PUFA—polyunsaturated fatty acids. MIX includes combined concentrations of C18:1 n9t + C18:1 n9c, C21:0 + C20:3 n6, and C24:1 n9 + C22:6 n3. Asterisks denote statistically significant differences at *p* ≤ 0.01. Error bars represent 95% confidence intervals. Abbreviations: Ac—acid derivatization; Al—alkaline derivatization.

**Figure 3:**
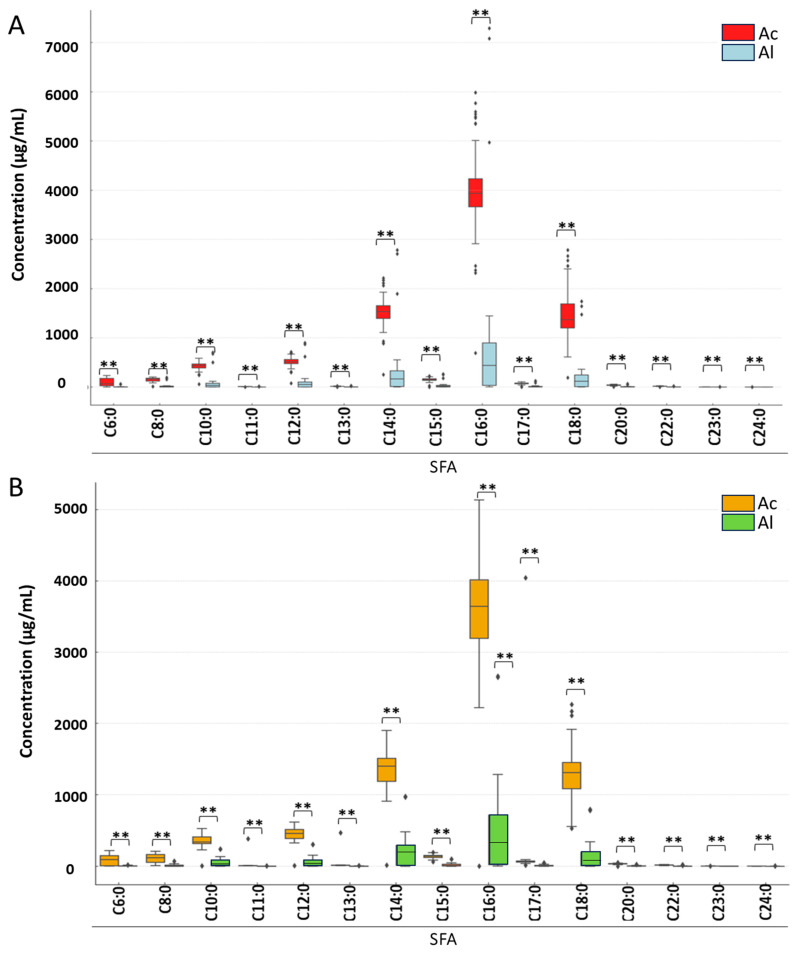
Changes in saturated fatty acid (SFA) concentrations depend on the type of derivatization and phase of pasteurization: (**A**) before and (**B**) after pasteurization. Asterisks denote statistically significant differences at *p* ≤ 0.01. Abbreviations: Ac—acid derivatization; Al—alkaline derivatization.

**Figure 4:**
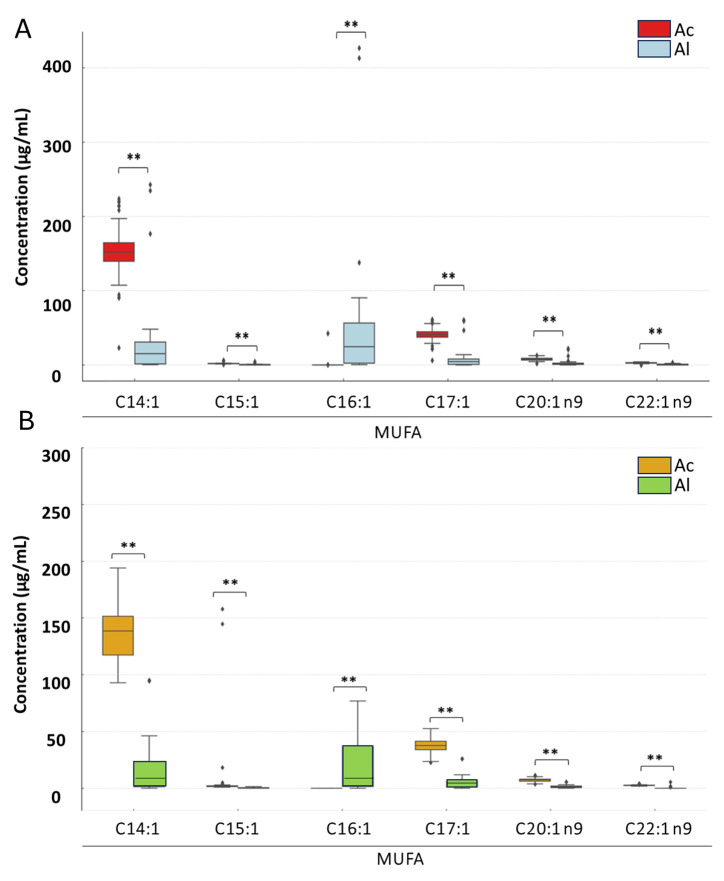
Changes in monounsaturated fatty acid (MUFA) concentrations depending on the type of derivatization and pasteurization phase: (**A**) before pasteurization and (**B**) after pasteurization. Asterisks denote statistically significant differences at *p* ≤ 0.01. Abbreviations: Ac—acid derivatization; Al—alkaline derivatization.

**Figure 5:**
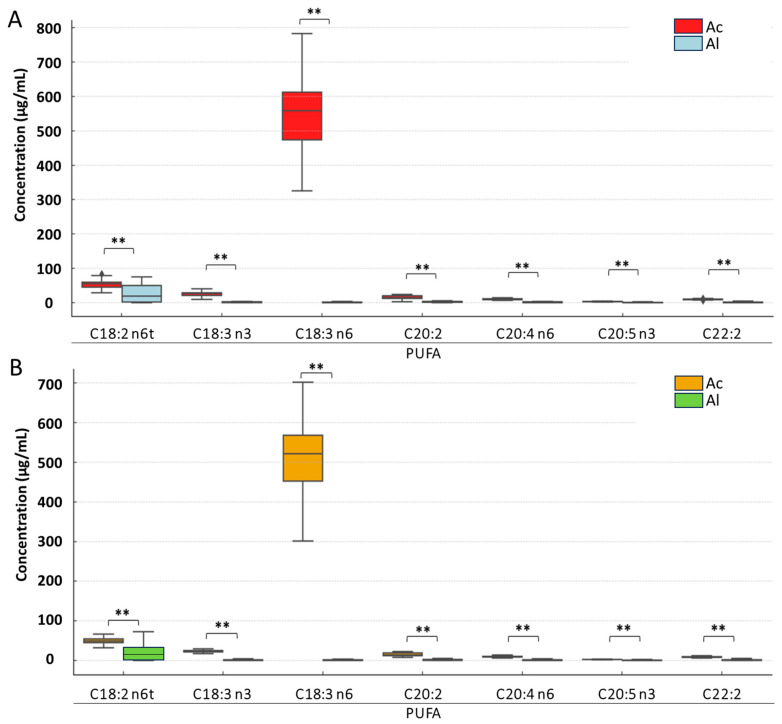
Changes in polyunsaturated fatty acid (PUFA) concentrations depending on the type of derivatization and pasteurization phase: (**A**) before pasteurization and (**B**) after pasteurization. Asterisks denote statistically significant differences at *p* ≤ 0.01. Abbreviations: Ac—acid derivatization; Al—alkaline derivatization.

A two-way ANOVA was conducted to assess a statistically significant effect of the pasteurization phase and derivatization type on FA concentrations. In addition, paired *t*-tests were applied to determine whether differences in FA concentrations between acid and alkaline derivatization methods were statistically significant (Figure 1 and Figure 2). The Mann–Whitney U test was applied to compare differences in FA concentrations between the two types of derivatizations within specific groups. Box-plot visualizations (Figure 3, Figure 4 and Figure 5) were created to present the median, interquartile range (IQR), and data range. To illustrate the percentage changes in the concentrations of three lipid classes following the pasteurization process, a heatmap was generated based on the identification of lipids after acid derivatization. The statistical significance threshold was set at *p* ≤ 0.05.

## 3. Results

Only by simultaneously considering the structure of the resulting derivatives, quantitatively assessing reaction efficiency, selecting the most favorable conditions for its execution, and directly comparing it with other procedures using the same real-world samples, can a method be developed to achieve results that are sufficiently reliable and comparable with data published by other researchers [20,21,22]. The most commonly used and one of the most effective techniques for analyzing FAs is GC with prior formation of methylated derivatives analyzed using GC–FID. The conversion of FAs into methyl esters occurs during the derivatization process, which imparts the compounds with properties necessary for GC analysis, such as reduced polarity, increased volatility, and improved thermal stability [23].

**Figure 6:**
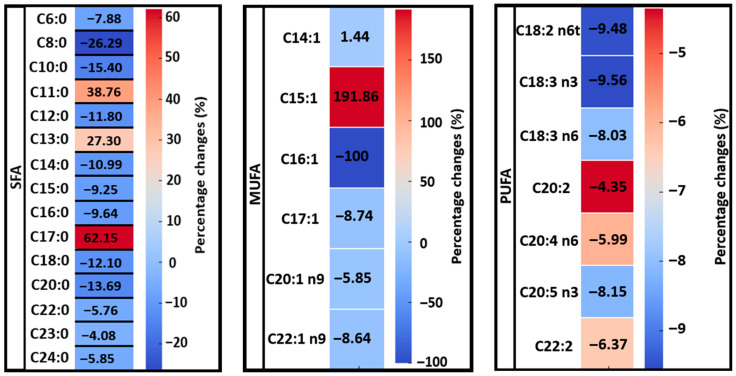
Heatmap representation of the percentage change in the concentration of three lipid classes: saturated fatty acids (SFA), monounsaturated fatty acids (MUFA), and polyunsaturated fatty acids (PUFA) after pasteurization and acid derivatization of the cream. The color scale reflects the direction and magnitude of the FAs concentration change: red hues indicate an increase, while blue hues indicate a decrease, relative to the pre-pasteurization levels. The intensity of the color corresponds to the percentage magnitude of the change.

The chromatographic conditions and adjusted temperature program used in this study allowed for the separation of FAs with varying molecular weights. Because of methylation, 31 out of 37 standard FAMEs yielded clear signals in the form of peaks, enabling their identification based on the retention times of individual compounds (Supplementary Figure S1 and Supplementary Table S1). The study was conducted to evaluate the effect of pasteurization and the derivatization method used on the concentration of FAs in cream samples (Figure 1, Figure 2, Figure 3, Figure 4, Figure 5 and Figure 6, Supplementary Figures S2 and S3).

### 3.1. The Effect of Derivatization and Pasteurization Methods on the Overall Concentration of Fatty Acids, Along with the Division into Specific Lipid Classes

The applied methodology allowed for the detection of 34 different FAs in the cream samples, of which 16 were saturated and the remaining 18 were unsaturated. Among them, 8 were identified as FAs with odd-numbered chains. The reference mixture did not contain FAs with branched chains, which prevented their identification and quantification. In the study by Machado et al. [24], GC analysis revealed the presence of 29 FAs. Cream samples were generally rich in SFAs, followed by MUFAs and a small percentage of PUFAs.

A two-way ANOVA revealed that both the pasteurization phase and the type of derivatization had statistically significant effects on FA concentrations (*p* = 0.027 and *p* < 0.001, respectively). However, no significant interaction between these two factors was observed. The type of derivatization was found to significantly influence the range of detectable FA concentrations, regardless of the pasteurization phase (Figure 1). Acid derivatization allowed for the determination of significantly higher FA concentrations compared to alkaline derivatization. Before pasteurization, the mean concentrations obtained were 302.26 µg/mL for acid derivatization and 62.66 µg/mL for alkaline derivatization. After pasteurization, the mean concentrations were 272.35 µg/mL and 41.20 µg/mL, respectively. Acid derivatization also provided a broader range of FA concentration variability.

The results of the two-way ANOVA for FA categories demonstrated that the type of derivatization had a statistically significant effect on the concentrations of all FA types (*p* < 0.001). In contrast, the pasteurization phase significantly influenced the concentrations of SFA and MIX of FA (*p* = 0.050 and *p* < 0.024, respectively) but had no significant effect on MUFAs or PUFAs. The interaction between the pasteurization phase and the type of derivatization was statistically significant only for the MIX category.

Considering both pre- and post-pasteurization stages, the range of FA concentrations within FA categories varied significantly depending on the derivatization method used (Figure 2). For cream samples collected prior to pasteurization, the mean concentration of SFAs obtained through acid derivatization was significantly higher (563.27 µg/mL) compared to alkaline derivatization (93.59 µg/mL). A similar trend was observed for MUFAs, with mean concentrations of 34.37 µg/mL and 12.48 µg/mL, respectively, for acid and alkaline derivatization, and for PUFAs, with mean concentrations of 95.43 µg/mL and 8.17 µg/mL, respectively. After pasteurization, acid derivatization also resulted in significantly higher mean concentrations of SFAs (505.49 µg/mL vs. 63.6 µg/mL), MUFAs (34.6 µg/mL vs. 9.91 µg/mL), and PUFAs (87.73 µg/mL vs. 7.28 µg/mL) compared to alkaline derivatization. In the study by Machado et al. [22] the main FAs of raw cream were C16:0 at the level of 24.50 ± 0.12%, C18:1c at the content of 20.03 ± 0.11% and C14:0 at the amount of 11.25 ± 0.07%, which is consistent with the literature data [25,26].

Our results suggest that acidic derivatization is a more effective method for accurately determining the concentrations of both saturated (SFAs) and unsaturated FAs compared to the alkaline method (Figure 2). Researchers from Ostermann et al. [27] compared various derivatization methods for FAs, including derivatization using HCl, boron trifluoride (BF3), KOH, trimethylsulfonium hydroxide (TMSH), and a combination of NaOH and BF3, analyzing various biological samples. Their findings showed that each method has limitations; for example, BF3 was ineffective for cholesterol and triacylglycerol (TAG) derivatization, while KOH derivatization led to incomplete derivatization of free FAs (FFAs). Furthermore, HCl derivatization and the combination of BF3 with NaOH were comparable for FFAs, polar lipids, and TAG derivatization. Kramer et al. [28] compared several acidic and alkaline derivatization methods for analyzing milk fats from ruminants and found that the best derivatization was achieved using sodium methoxide (NaOCH_3_), followed by HCl, BF3, or diazomethane. Multi-step methods combining acid- and base-catalyzed methylation have been widely adopted for total FA analysis in milk [29]. Another reagent, tetramethylammonium hydroxide (TMAH), can instantly convert FFAs to FAMEs or form FFA salts in separate phases, allowing both lipid extract components to be analyzed without prior separation [30]. However, TMAH has limitations since the glyceride component of lipid extracts interferes with FFA determination [16]. To determine very long-chain FAs by GC, Antolín et al. [31] examined five derivatization methods using diazomethane, H_2_SO_4_, HCl, BF3, and TMSH. Among them, the HCl method required more time for derivatization completion. Considering cost, speed, safety, and GC principles, the H_2_SO_4_ method was deemed most suitable for very long-chain FAs. In another study, introducing trimethylsilyldiazomethane (TMSDM) or HCl after alkaline catalysis was effective for complete FFA methylation, providing satisfactory results for FA composition, including total FA, in margarine samples [32]. Comparing the same extraction method, i.e., microwave-assisted hydrolysis extraction and microwave-assisted solvent extraction, with two different derivatization procedures (HCl/MeOH and BF3), no differences were observed for any matrix or FAME. From the derivatization perspective, the overhyped BF3 derivatization can be successfully replaced with HCl/MeOH microwave-assisted derivatization, achieving similar efficiency while improving operator safety by using less harmful reagents, being generally more environmentally friendly, and providing higher throughput [33].

### 3.2. Analysis of Detailed Changes in Individual Fatty Acid Classes Under Derivatization and Pasteurization

Our analyses also allowed us to interpret in detail the different types of lipids in identyfied lipids class (SFA, MUFA, PUFA) in the cream samples (Figure 3, Figure 4, Figure 5 and Figure 6).

The two-way ANOVA results revealed a variable statistical significance of the pasteurization phase depending on the specific FA analyzed (Supplementary Table S2). The pasteurization phase significantly influenced the concentrations of FAs such as C8:0, C10:0, C11:0, C12:0, C14:0, C15:0, C16:0, C18:0, and C20:0. However, no significant effect of pasteurization was observed for FAs including C6:0, C13:0, C17:0, C22:0, C23:0, and C24:0. The type of derivatization had a statistically significant impact on the concentrations of all identified SFA. The interaction between the pasteurization phase and the type of derivatization was statistically significant only for C8:0 and C10:0, while for the majority of FAs, this interaction was not significant.

The concentrations of SFA identified in cream before pasteurization differed significantly between the two types of derivatization (Figure 3). In all analyzed cases, significantly higher SFAs concentrations were observed following acid derivatization. The largest differences in concentration between the two derivatization methods were found for C6:0, C8:0, C18:0, and C22:0. Regardless of the derivatization method, the highest mean concentration was observed for C16:0 (3966.86 µg/mL for acid derivatization and 717.41 µg/mL for alkaline derivatization), while the lowest was for C24:0 (1.39 µg/mL and 0.35 µg/mL, respectively).

Similarly, SFA concentrations in cream after pasteurization also showed statistically significant differences between the two derivatization methods for all identified SFAs. Significantly higher concentrations were observed for each identified SFA following acid derivatization. Consistent with the pre-pasteurization results, the highest mean concentration post-pasteurization was noted for C16:0 (3966.86 µg/mL and 717.41 µg/mL for acid and alkaline derivatization, respectively), and the lowest was observed for C24:0 (1.39 µg/mL and 0.35 µg/mL, respectively).

The results of the two-way ANOVA revealed that the pasteurization phase had a statistically significant effect on the concentrations of only two identified MUFA, i.e., C17:1 and C20:1 n9 (*p* = 0.004 and *p* = 0.017, respectively). For all identified MUFAs, the type of derivatization had a statistically significant effect on their concentrations. No statistically significant interaction was observed between the pasteurization phase and the type of derivatization for any of the identified MUFAs.

MUFA concentrations in cream before pasteurization differed significantly between the two derivatization methods (Figure 4). The highest mean concentrations were observed for C14:1 (151.83 µg/mL and 40.82 µg/mL, respectively, for acid and alkaline derivatization), while the lowest was noted for C22:1 n9 (2.83 µg/mL and 0.41 µg/mL, respectively, for acid and alkaline derivatization). In nearly all analyzed MUFAs, significantly higher concentrations were detected following acid derivatization. The largest differences between the two methods of derivatization were observed for C14:1. Interestingly, for C16:1, alkaline derivatization resulted in a 70-fold higher concentration compared to acid derivatization (40.82 µg/mL vs. 0.58 µg/mL, respectively).

Post-pasteurization, the highest mean concentration was again observed for C14:1 following acid derivatization (154.02 µg/mL) and for C16:1 following alkaline derivatization (36.82 µg/mL). The lowest concentrations were noted for C15:1 and C22:1 n9 after alkaline derivatization (0.35 µg/mL and 0.37 µg/mL, respectively). Notably, after pasteurization, C16:1 was not detected in the acid-derivatized sample, although it was identified in the corresponding alkaline-derivatized sample. Similar to the pre-pasteurization results, significantly higher concentrations were detected for nearly all analyzed MUFAs following acid derivatization, with the largest differences observed for C14:1, C17:1.

The results of the two-way ANOVA revealed that the pasteurization phase had a statistically significant effect on the concentrations of C18:3 n3, C18:3 n6, C20:4 n6, and C20:5 n3 from the PUFA group. For all identified PUFAs, the type of derivatization had a statistically significant effect on their concentrations. No statistically significant interaction was observed between the pasteurization phase and the type of derivatization for any of the identified PUFAs.

PUFA concentrations in cream prior to pasteurization differed significantly between the two derivatization methods (Figure 5). The highest mean concentrations were observed for C18:3 n6 (553.73 µg/mL and 1.07 µg/mL, respectively, for acid and alkaline derivatization), while the lowest concentrations were noted for C20:5 n3 (3.0 µg/mL and 0.7 µg/mL, respectively, for acid and alkaline derivatization). For all analyzed PUFAs, significantly higher concentrations were detected following acid derivatization, with the largest difference between the two methods observed for C18:3 n6.

After pasteurization, the highest mean PUFA concentrations in cream were again observed for C18:3 n6 (508.6 µg/mL and 1.12 µg/mL, respectively, for acid and alkaline derivatization), while the lowest were for C20:5 n3 (2.79 µg/mL and 0.61 µg/mL, respectively, for acid and alkaline derivatization). As with the pasteurization results before, significantly higher concentrations were observed for all analyzed PUFAs following acid derivatization, with the largest difference between the two derivatization methods again noted for C18:3 n6.

The FAs detected in this study in the cream samples may also be synthesized by bacteria living in the rumen of cows [34,35]. A small amount may also be produced in the further sections of the digestive tract, e.g., some linear C15:0 and C17:0 FAs can be obtained through de novo synthesis in adipocytes using propionyl-CoA as a precursor. FAs with branched chains may also originate from the metabolism of other biomolecules, including branched-chain amino acids (BCAA), such as valine, leucine, and isoleucine. These branched FAs are primarily produced by anteiso-amylolytic and isocellulolytic bacteria [36]. Odd-chain fatty acids are considered beneficial to human health, reducing the risk of cardiovascular diseases and type II diabetes, while branched-chain FAs exhibit potential anticancer properties. Short-chain fatty acids (SCFAs), with fewer than 6 carbon atoms, serve as an energy source, allowing them to provide energy for the growth and metabolism of colon epithelial cells. They may also regulate the human gut microbiota, influencing the secretion and activity of certain bacterial enzymes. Similarly, SCFAs and some medium-chain FAs may be involved in processes such as inhibition of cancer cell growth, proliferation, and promoting apoptosis in skin tissues [37].

The intensity of percentage (%) changes in the concentrations of individual FAs within the SFA, MUFA, and PUFA groups as a result of the pasteurization process was identified (Figure 6). For SFA, the largest decreases were noted for C8:0 and C10:0, with reductions of 26.29% and 15.40%, respectively, after pasteurization. Conversely, the concentrations of C17:0, C11:0, and C13:0 increased after pasteurization by 62.15%, 38.76%, and 27.30%, respectively.

In the case of MUFAs, a complete reduction in the concentration of C16:1 was observed, along with slight decreases in the concentrations of C17:1, C20:1 n9, and C22:1 n9 following pasteurization. However, the concentration of C15:1 nearly tripled, showing a 191.86% increase compared to its pre-pasteurization level.

For all identified PUFAs, decreases in concentrations were observed after pasteurization. The largest reductions were noted for C18:2 n6t, C18:3 n3, C20:5 n3, and C18:3 n6, with percentage decreases of 9.48%, 9.56%, 8.15%, and 8.03%, respectively.

In the study by Tormási and Abrankó [38] the cream had a fat content of 18.9 ± 0.75% and according to their GC-FID analysis it contained the following amounts of FAs: C16:0 at the level of 32.4 ± 0.1%, C18:1n-9c at the level of 23.3 ± 0.1%, C14:0 at the level of 11.7 ± 0.1%, C18:0 at the value of 10.8 ± 0.1%, C12:0 at the level of 3.8 ± 0.2%, C18:2n-6c at the level of 3.5 ± 0.1%, C10:0 at the level of 3.4 ± 0.3%, C16:1n-7c at the level of 2.0 ± 0.3%, C18:1n-9c at the level was 1.8 ± 0.3%, and C15:0 was 1.2 ± 0.02%. The reported FA composition of the tested cream was consistent with the literature data [39]. Furthermore, different processing methods did not affect (*p* > 0.05) SFA and MUFA, while the level of PUFA decreased (*p* ≤ 0.05) on the 52nd day of storage. Regarding the main FAs in cream, only C16:0 was present in higher amounts (*p* ≤ 0.05) in raw cream (compared to processed samples) [24]. Moltó-puigmartí et al. [40] reported that high-pressure processing (HPP) did not significantly change the FAs proportion compared to unprocessed human milk.

## 4. Discussion

These results are crucial for understanding the biochemical processes occurring in milk samples and for evaluating the quality of these products. The lack of significant changes in the FA composition may indicate the stability of fats in the analyzed samples, which is a positive characteristic from the standpoint of the durability and quality of dairy products. The stability of milk fats suggests that they can be used in various other bioformulations without the risk of rapid lipid breakdown, which could affect the quality and shelf life of cosmetics. Furthermore, the stability of milk fats may allow for longer storage of certain products without the need for artificial substances, chemical additives, stabilizers, etc., which is in line with the trend toward natural and minimally processed bioproducts. This may attract consumers looking for more natural and environmentally friendly options.

Milk and dairy products have been a cornerstone of human nutrition for millennia and represent one of the most important agricultural commodities for human nourishment. Dairy products are included in most dietary guidelines worldwide as they supply a variety of essential nutrients and several key components that are often deficient in the human body. Motivated by the utilitarian significance of dairy products in the human diet, regarded as the richest source of natural bioactive components [41,42], we decided to analyze the FA profile in cream. Sour cream is a popular fermented dairy product with high fat content—minimum 10% fat content [43]. Sour cream is produced from only two key ingredients: cream (containing about 30% milk fat) and LAB cultures. The production steps include standardization, pasteurization, homogenization and fermentation with the addition of bacterial cultures. All these steps are responsible for shaping the product structure. Heat treatment leads to denaturation of proteins surrounding milk fat globules (MFG) as well as serum proteins [44] and facilitates interactions between denatured MFGM and milk serum proteins [45]. Milk cream is a component of many products such as butter, ice cream, and sour cream [3]. However, it is a highly perishable product with a pH and high water activity, requiring proper preservation to extend its shelf life [46]. Traditionally, most cream intended for retail and industrial applications undergoes thermal pasteurization aimed at destroying vegetative microorganisms (pathogenic and spoilage-causing) and deactivating enzymes, thereby extending product longevity [47,48]. However, depending on the food matrix, thermal pasteurization may not always be ideal as it can cause significant changes in optimal product quality, such as undesirable flavors and loss of vitamins and other minerals. Consumers place great importance on the texture, flavor, aroma, shape, and color of food, and there is growing demand for minimally processed and long-lasting products. Consequently, alternative preservation methods, especially non-thermal ones that retain the sensory and nutritional properties of food, are being tested and developed [49].

Our analysis results showed that pasteurization had varying effects on different lipid classes and their components (Figure 3, Figure 4, Figure 5 and Figure 6), indicating potential chemical changes in lipid structures due to temperature. Different FAs responded to pasteurization in different ways, highlighting the complexity of chemical processes occurring during heat treatment. For instance, C15:1 and C17:0 acids showed increased concentrations after pasteurization, possibly due to their release from more complex lipid structures (Figure 4). Furthermore, pasteurization significantly reduced overall FA concentrations, particularly for SFAs (Figure 3), likely due to chemical reactions such as oxidation and isomerization during heat treatment. Most MUFAs and PUFAs also decreased post-pasteurization (Figure 5 and Figure 6), indicating that the thermal process affects both groups. A decrease in C18:2 n6t and C20:5 n3 concentrations was noted after pasteurization, as these are particularly susceptible to thermal degradation (Figure 6). Conversely, Figure 6 shows the grouping of FA classes, where SFAs were more stable compared to PUFAs, which were more prone to changes due to pasteurization. In studies by Machado et al. [24], GC analysis revealed the presence of 29 FAs in raw cream. Samples were primarily rich in SFAs, followed by MUFAs, with a small percentage of PUFAs. Major FAs in raw cream were C16:0, C18:1c, and C14:0, consistent the findings of other researchers [25,26,50].

Our results show that the acid-catalyzed method (following hexane extraction) was more effective. Hexane extraction was sufficient for cream—cream is primarily TAG. The hexane-only extraction step could recover the largest amount of lipids. The pure hexane extract was then subjected to very effective acid catalysis. Regarding the efficiency of base derivatization, the chloroform/methanol extract contained the entire lipid pool, including phospholipids, water, and free fatty acids. Base catalysis is extremely sensitive to saponification (hydrolysis) and moisture. The presence of a high phospholipid content in this extract likely inhibited the reaction, leading to very low FAME measurements. Although hexane extraction may be less efficient, the acid derivatization we used is sufficiently robust to convert the recovered hexane to FAME with high efficiency (Figure 1, Figure 2, Figure 3, Figure 4, Figure 5 and Figure 6). Moreover, acidic conditions can lead to the migration of double bonds in the fatty acid chain, creating geometric or positional isomers that change properties. For example, unstable double bonds in PUFAs are highly susceptible to oxidation, especially under harsh reaction conditions such as high temperatures or strong acids. Alkaline-catalyzed methods, while generally milder, may not completely convert all FFAs to their esterified forms, leading to an underestimation of their total amount [51,52,53]. The study by Sutherland [54] for the first time comprehensively compares four derivatization procedures (TMTFTH, NaOEt–BSTFA, KOH–BSTFA, and ACM) for the analysis of fatty acid composition in oils. Three types of oils (analytical-grade rapeseed oil, linseed oil, commercial rapeseed oil) and a mixture of TAGs with known content were used for quantitative validation. TMTFTH proved to be the best method for the quantitative analysis of fatty acids, achieving the highest efficiency 96 ± 2% and the best repeatability. KOH–BSTFA also has high efficiency 95 ± 7%, but it is a two-step procedure (longer time) and shows poorer repeatability. The main drawback is the necessity of synthesizing commercially unavailable TMSE standards. ACM is less efficient 83 ± 3%, and although it uses readily available reagents, it is labor-intensive and time-consuming. NaOEt–BSTFA was the least efficient 64 ± 2% and is also a two-step procedure. Conversely, the study by Topolewska et al. [55] revealed that the most effective method for analyzing plant fatty acids using GC is derivatization with (trimethylsilyl)diazomethane (TMSD). This method is more accurate than the standard BF3/MeOH reaction and generates fewer interferences in the critical retention time range. A drawback, however, is the possibility of overestimating the FA(18:0) content when using TMSD. Derivatization methods using trimethylsilyl or tert-butyldimethylsilyl derivatives proved to be more difficult and unreliable for complete analysis due to transesterification products, poor separation of FA(18:X) compounds, and a high level of interference. They may only be an alternative for GC-MS analysis when full separation is not required, but further research is necessary.

Milk fat consists of an impressive FA composition, containing over 400 different FAs and their derivatives. This is responsible for numerous nutritional, organoleptic, and technological properties of milk and dairy products [56]. It contains a unique diversity of bioactive FAs, which are synthesized by microorganisms found in the rumen of animals and in the mammary glands [57]. This fat consists of short- and medium-chain FAs, positional and geometric isomers of octadecanoic acid (18:1), conjugated linoleic acids, FAs with odd chain lengths (15:0 and 17:0), and branched-chain FAs (BCFA). Among the FAs in milk fat, about 14% are unique FAs derived from milk, and several of these have an impact on the normal physiology of mammals, acting as bioactive molecules that exert beneficial properties to support health and prevent chronic diseases [58,59,60]. In particular, BCFA, which make up about 2% of milk fat, have recently been identified as bioactive molecules contributing to the positive health effects associated with the consumption of full-fat dairy products [61]. The presence of different secondary fatty acids (FAs) is of great importance because they contribute to the diverse triacylglycerol profile in milk fat, meaning that many TAGs with different intramolecular structures will be present in milk fat. In the case of TAGs with different intramolecular compositions, the possibility of different regioisomers further increases this diversity. The nutritional significance of the high FA content and the resulting TAG diversity in milk fat lies in the fact that the intramolecular structure of TAGs influences the digestibility of dietary FAs, as lipase enzymes may have different preferences for TAGs with different structures [44,62,63].

Additionally, fortification of cream, i.e., enriching it with extra vitamins (e.g., vitamins D_3_) and modifying the FA composition, is crucial for improving the health value of dairy products, as it has a significant impact on enhancing the lipid profile of the consumer’s diet. This process provides greater amounts of healthy fats, such as MUFAs, while reducing the amount of SFAs, which may support cardiovascular health [64]. Moreover, the addition of probiotic bacteria, such as *Lactobacillus plantarum*, allows for the natural biosynthesis of beneficial components in dairy products [65]. This process is especially important in regions where diets are deficient in vitamins and exposure to sunlight is limited, increasing the risk of vitamin D deficiencies [66]. By fortifying cream, it is possible to deliver valuable nutrients in the daily diet, which supports overall health and may help prevent many diseases. This is perfectly confirmed by a recent study we conducted, where the addition of the *L. plantarum* B/00401 strain to cream enhanced vitamin D_3_ biosynthesis and altered the FA composition, with a trend toward reduced SFA and increased MUFA following incubation with *L. plantarum* B/00401 [67].

## 5. Conclusions

Based on the analyses, it can be concluded that the pasteurization process has a varied effect on the FA profile of the analyzed cream, indicating the complexity of the chemical reactions occurring under the influence of temperature. Pasteurization reduces the overall content, especially SFA, and influences changes in the content of MUFA and PUFA. There is a noticeable increase in the concentration of unusual chain lengths (e.g., C15:1, C17:0), which may indicate their release from complex lipid structures, while the concentrations of all decrease after pasteurization. The analyses confirm the importance of lipid quality in dairy products, including cream, and suggest potential health benefits resulting from minimal processing, which helps maintain the beneficial organoleptic and nutritional properties of these products. This study showed that the derivatization method significantly affects the obtained quantitative results: acid derivatization (Ac) is more efficient and provides considerably higher overall concentrations (e.g., before pasteurization: 302.26 µg/mL for Ac vs. 62.66 µg/mL for the alkaline method), making it recommended for the accurate determination of FA in cream. The alkaline method consistently gave lower concentrations, although in one case (C16:1) it showed higher efficiency before pasteurization.

## Data Availability

The original contributions presented in this study are included in the article/Supplementary Material. Further inquiries can be directed to the corresponding author.

## Supplementary Materials

The following supporting information can be downloaded at: https://www.mdpi.com/article/10.3390/foods14223815/s1, Figure S1: Chromatogram from GC-FID analysis of fatty acids methyl esters (standard) (Supelco 37 Component FAME Mix; Sigma-Aldrich, Schnelldorf, Germany); Figure S2: Chromatograms from GC-FID analysis of fatty acids from cream: (A) before pasteurization using acid derivatization, (B) after pasteurization using acid derivatization; Figure S3: Chromatograms from GC-FID analysis of fatty acids from cream: (A) before pasteurization using acid derivatization, (B) after pasteurization using alkali derivatization; Table S1: Physicochemical parameters of 30 cream samples before and after pasteurization. Parameters such as acidity in Soxhlet-Henkel [°SH] degrees, pH value, as well as fat, protein, lactose and dry matter content expressed in % were determined; Table S2: The patterns of the identified fatty acids, their retention time and the coefficient of determination of the calibration curves (R^2^); Table S3: Results of the two-way analysis of variance (ANOVA) conducted for two factors: Factor 1 (pasteurization phases), Factor 2 (type of derivatization), and the interaction effect between these factors. Statistically significant results (*p* < 0.05) are highlighted in orange in the table.

## Abbreviations

FAs	Fatty Acids
FAME	Fatty Acid Methyl Esters
GC-FID	Gas Chromatography-Flame Ionization Detector
SFA	Saturated Fatty Acids
MUFA	Monounsaturated Fatty Acids
PUFA	Polyunsaturated Fatty Acids
