# Supplementary material for: Revealing the Impact of Pasteurization and Derivatization Chemistry on the Fatty Acid Profile of Dairy Cream: A Comparative Approach
Source: Foods. 2025 Nov 7;14(22):3815. doi: 10.3390/foods14223815 (PMC12650865; doi:10.3390/foods14223815)
Supplement: Supplementary file 1 [file foods-14-03815-s001.zip › foods-3907653-supplementary.pdf]

SUPPLEMENTARY MATERIALS

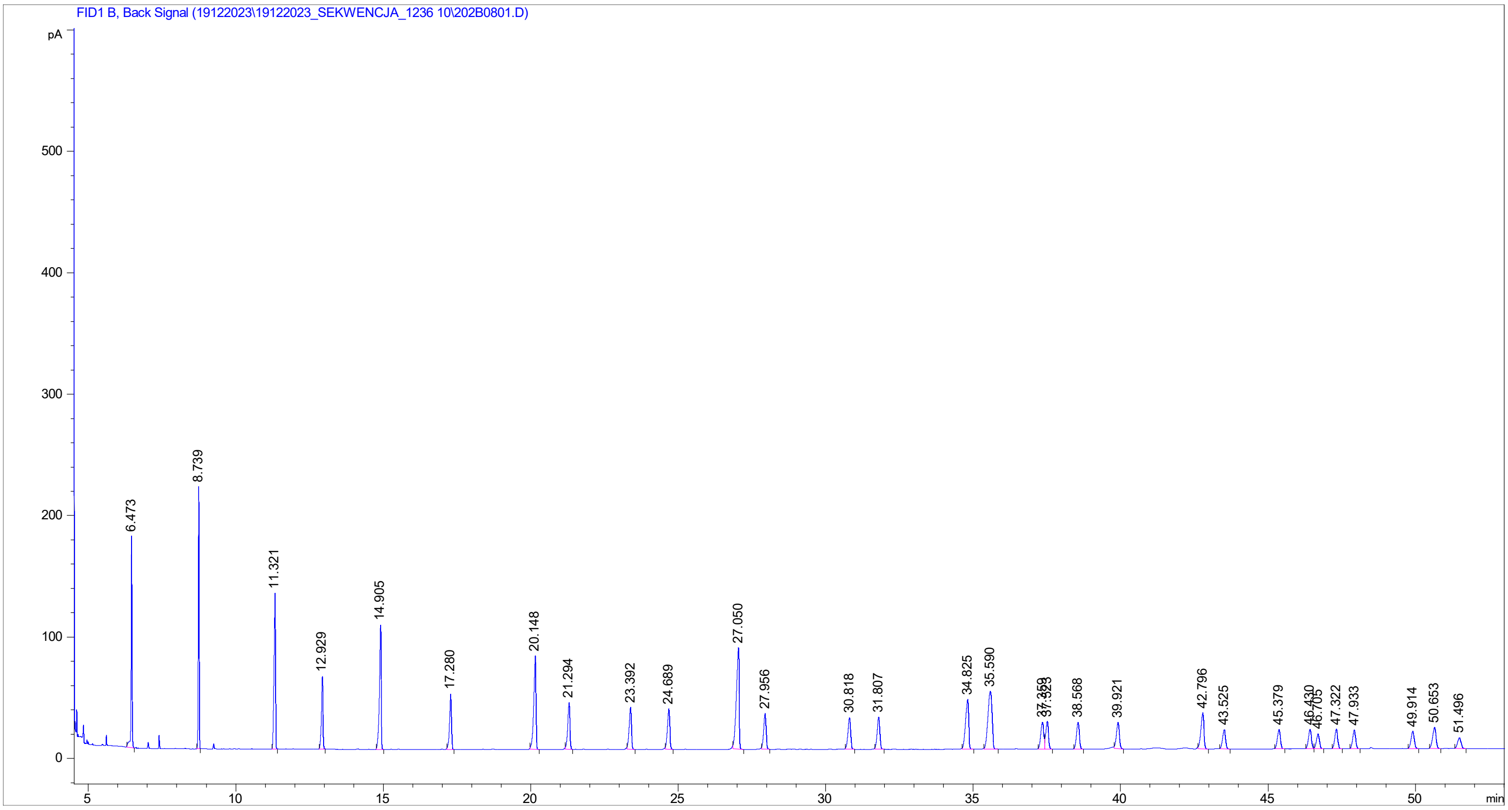

Supplementary Figure S1. Chromatogram from GC-FID analysis of fatty acids methyl esters (standard) (Supelco 37 Component FAME Mix; Sigma-Aldrich, Schnelldorf, Germany).

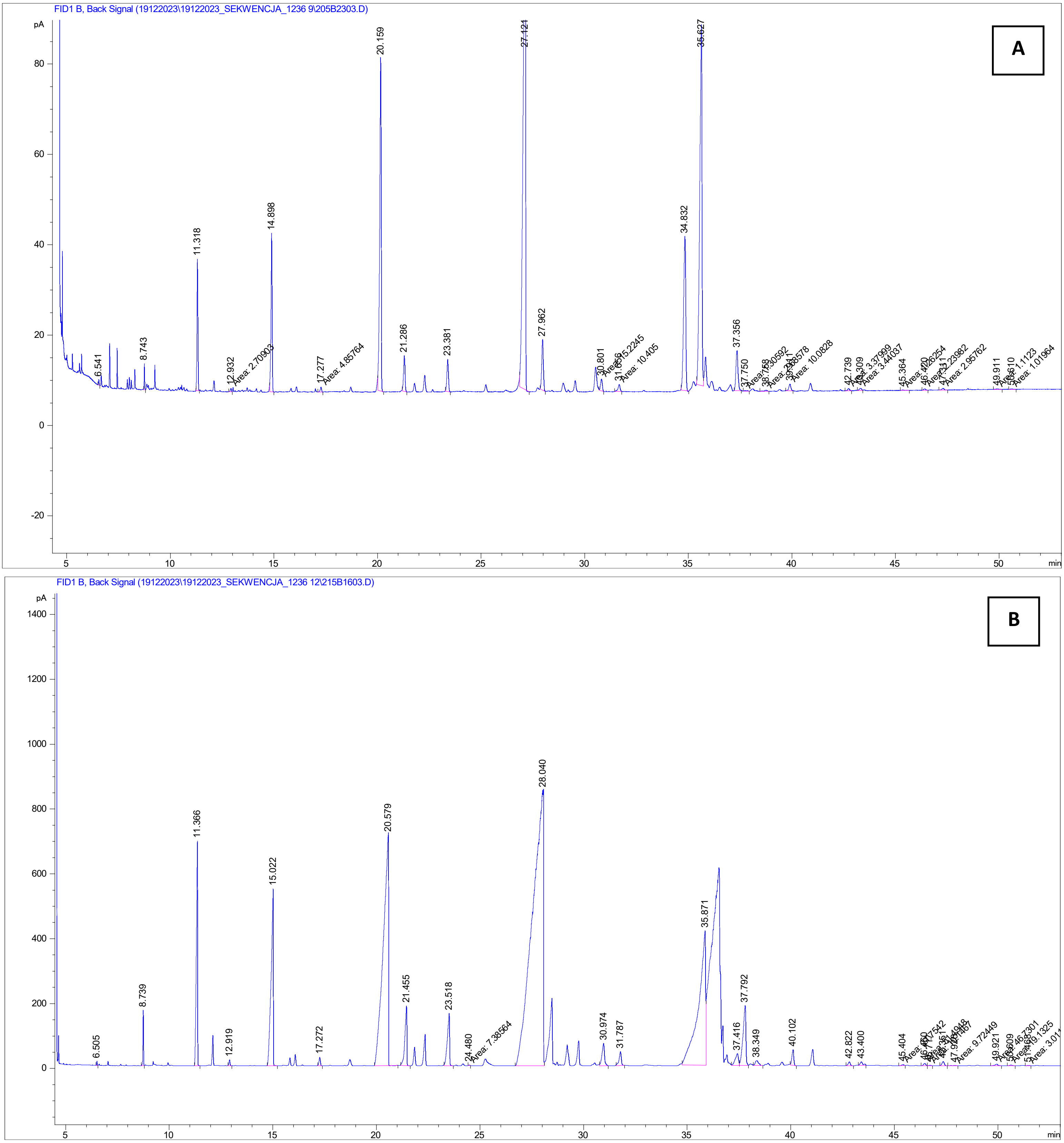

Supplementary Figure S2. Chromatograms from GC-FID analysis of fatty acids from cream: (A) before pasteurization using acid derivatization, (B) after pasteurization using acid derivatization.

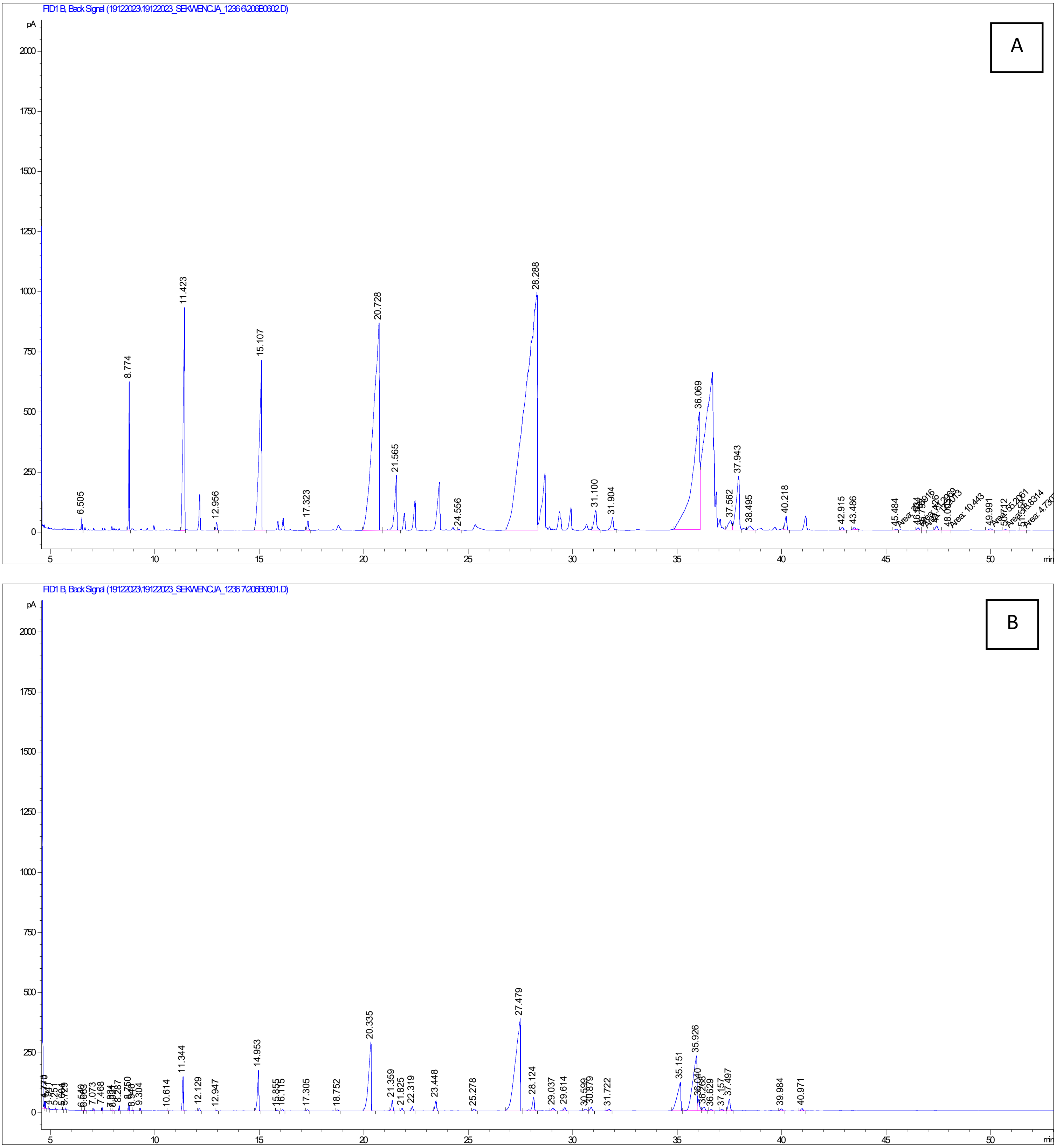

Supplementary Figure S3. Chromatograms from GC-FID analysis of fatty acids from cream: (A) before pasteurization using acid derivatization, (B) after pasteurization using alkali derivatization.

Supplementary Table S1. Physicochemical parameters of 30 cream samples before and after pasteurization. Parameters such as acidity in Soxhlet-Henkel [°SH] degrees, pH value, as well as fat, protein, lactose and dry matter content expressed in % were determined.

Physicochemical properties of cream before pasteurization						
No.	°SH	pH	Fat	Protein	Lactose	Dry matter
			[%]			
1	4.60	6.72	41.00	1.58	2.34	44.12
2	4.73	6.69	41.00	1.70	2.51	44.45
3	4.87	6.69	38.50	1.66	2.44	42.66
4	5.00	6.68	40.50	1.73	2.40	45.17
5	4.87	6.66	41.00	1.74	2.44	44.64
6	5.60	5.95	39.38	2.00	2.70	33.77
7	5.60	6.67	40.01	1.98	2.67	45.34
8	5.60	6.67	39.49	1.99	2.69	44.86
9	5.60	6.67	39.77	1.99	2.69	45.11
10	5.60	6.66	40.12	1.98	2.67	45.44
11	5.20	6.70	39.50	1.85	2.59	49.19
12	5.40	6.69	41.00	1.76	2.45	44.90
13	5.00	6.73	39.50	1.85	2.60	43.90
14	5.20	6.69	39.00	1.67	2.45	42.20
15	5.00	6.70	42.00	1.65	2.42	45.50
16	5.00	6.70	40.07	1.67	2.47	43.30
17	5.40	6.70	39.00	1.85	2.57	42.97
18	5.20	6.71	38.00	1.65	2.44	41.54
19	5.40	6.68	41.10	1.79	2.46	44.70
20	5.20	6.7	40.20	1.73	2.42	44.53
21	5.00	6.72	40.50	1.79	2.55	44.55
22	5.00	6.71	40.50	1.80	2.56	44.71
23	5.20	6.68	42.00	1.69	2.44	45.31
24	5.00	6.71	40.50	1.70	2.50	44.25
25	5.00	6.71	40.50	1.70	2.52	44.28
26	5.00	6.70	41.00	1.75	2.44	44.62
27	5.20	6.70	42.00	1.73	2.43	45.39
28	5.20	6.67	41.00	1.66	2.35	45.39
29	5.00	6.70	40.50	1.73	2.46	44.31
30	5.00	6.65	41.00	1.63	2.41	44.27
**Physicochemical properties of cream after pasteurization**						
No.	°SH	pH	Fat	Protein	Lactose	Dry matter
			[%]			
1	5.00	6.66	40.70	2.24	2.08	45.07
2	5.00	6.68	40.72	2.25	2.02	45.03
3	5.00	6.68	40.84	2.26	2.02	45.10
4	5.00	6.68	50.91	2.25	2.01	45.15
5	5.00	6.68	40.79	2.25	2.02	45.13
6	4.80	6.64	39.84	2.23	2.01	44.04
7	4.80	6.65	40.20	2.23	2.00	44.44
8	4.80	6.65	36.99	2.23	2.00	44.57
9	4.80	6.65	40.43	2.23	2.00	44.66

10	4.80	6.65	40.43	2.23	2.01	44.72
11	5.20	6.61	41.29	2.24	1.97	45.54
12	5.20	6.62	41.29	2.23	1.98	45.52
13	5.20	6.63	41.32	2.24	1.98	45.56
14	5.20	6.63	41.31	2.24	1.98	45.54
15	5.20	6.63	41.33	2.24	1.98	45.42
16	5.00	6.63	40.21	2.23	1.99	44.40
17	5.00	6.63	40.22	2.23	2.00	44.43
18	5.00	6.64	40.11	2.24	1.99	44.35
19	5.00	6.62	40.22	2.24	1.99	44.44
20	5.00	6.63	40.22	2.24	2.00	44.45
21	5.20	6.60	40.85	2.29	2.08	45.33
22	5.20	6.61	40.84	2.30	2.09	45.35
23	5.20	6.61	40.93	2.30	2.09	45.40
24	5.20	6.61	40.83	2.29	2.09	45.37
25	5.20	6.60	40.75	2.28	2.08	45.05
26	4.80	6.65	40.80	2.21	1.97	45.04
27	4.80	6.65	40.85	2.20	1.96	45.12
28	4.80	6.65	40.88	2.20	1.96	45.10
29	4.80	6.65	40.83	2.21	1.96	45.14
30	4.80	6.65	40.87	2.20	1.95	45.16

Supplementary Table S2. The patterns of the identified fatty acids, their retention time and the coefficient of determination of the calibration curves (R^2).

No.	Formula	Retention Time [min]	Calibration curve formula	Coefficient of determination (R^2)
1	C6:0	6.48	y = 9.7444x - 8.5533	R^2 = 0.9998
2	C8:0	8.74	y = 10.434x + 4.1655	R^2 = 0.9999
3	C10:0	11.32	y = 10.62x + 3.8981	R^2 = 0.9999
4	C11:0	12.928	y = 10.505x + 0.1805	R^2 = 0.9999
5	C12:0	14.903	y = 10.42x + 0.4809	R^2 = 1
6	C13:0	17.282	y = 10.238x - 1.6283	R^2 = 1
7	C14:0	20.149	y = 10.092x + 0.0755	R^2 = 1
8	C14:1	21.292	y = 9.876x - 2.6029	R^2 = 1
9	C15:0	23.391	y = 9.7125x - 2.2469	R^2 = 1
10	C15:1	24.689	y = 9.5485x - 3.0598	R^2 = 1
11	C16:0	27.051	y = 9.3492x + 5.8239	R^2 = 0.9999
12	C16:1	27.953	y = 8.8027x - 3.0373	R^2 = 1
13	C17:0	30.818	y = 8.6078x - 3.2507	R^2 = 0.9999
14	C17:1	31.808	y = 8.722x - 3.6191	R^2 = 1
15	C18:0	34.821	y = 7.8616x - 1.32	R^2 = 0.9999
16	C18:1 n9t + C18:1 n9c	35.601	y = 8.0749x - 6.1324	R^2 = 0.9999
17	C18:2 n6t	37.357	y = 8.6307x - 6.8064	R^2 = 0.9997
18	C18:3 n6	37.521	y = 3.754x - 2.952	R^2 = 1
19	C18:3 n3	38.568	y = 8.0376x - 5.8707	R^2 = 0.9999
20	C20:0	39.923	y = 9.9712x - 14.67	R^2 = 0.9989
21	C20:1 n9	42.799	y = 11.262x - 5.7077	R^2 = 0.9999
22	C20:2	43.523	y = 6.013x - 4.496	R^2 = 0.9998
23	C21:0 + C20:3 n6	45.383	y = 3.035x - 4.8079	R^2 = 0.9998
24	C20:4 n6	46.437	y = 6.1509x - 5.065	R^2 = 0.9998
25	C20:5 n3	46.707	y = 4.6498x - 3.0195	R^2 = 0.9999
26	C22:0	47.321	y = 6.3753x - 7.4541	R^2 = 0.9995
27	C22:1 n9	47.934	y = 5.8464x - 5.0917	R^2 = 0.9998
28	C22:2	49.918	y = 6.078x - 7.2377	R^2 = 0.9995
29	C23:0	50.659	y = 7.5759x - 5.7313	R^2 = 0.9998
30	C24:0	51.501	y = 3.9661x - 2.3677	R^2 = 0.9997
31	C24:1 n9 + C22:6 n3	53.699	y = 2.015x - 3.3836	R^2 = 0.9997

Supplementary Table S3. Results of the two-way analysis of variance (ANOVA) conducted for two factors: Factor 1 (pasteurization phases), Factor 2 (type of derivatization), and the interaction effect between these factors. Statistically significant results ($p < 0.05$) are highlighted in orange in the table.

Trait	**Factor 1** (pasterization phases)		**Factor 2** (type of derivatization)		Interaction effect	
	F	p	F	p	F	p
FA	4.897	0.027	410.947	p<0.001	0.132	0.716
SFA	3.825	0.050	411.867	p<0.001	0.382	0.536
MUFA	0.186	0.666	74.214	p<0.001	0.269	0.604
PUFA	0.564	0.452	214.22	p<0.001	0.353	0.055
MIX	5.137	0.024	36.251	p<0.001	4.389	0.036
C6:0	0.533	0.465	196.237	p<0.001	0.235	0.628
C8:0	25.726	p<0.001	702.569	p<0.001	14.957	p<0.001
C10:0	16.866	p<0.001	1073.580	p<0.001	5.082	0.024
C11:0	0.454	0.500	17.560	p<0.001	0.733	0.392
C12:0	13.624	p<0.001	1055.162	p<0.001	1.156	0.283
C13:0	0.325	0.568	27.889	p<0.001	0.804	0.371
C14:0	10.593	0.001	970.446	p<0.001	1.106	0.293
C15:0	9.324	0.002	978.688	p<0.001	0.284	0.593
C16:0	8.874	0.003	937.913	p<0.001	0.503	0.478
C17:0	0.649	0.421	10.145	0.002	0.826	0.364
C18:0	8.272	0.004	910.38	p<0.001	2.335	0.128
C20:0	15.654	p<0.001	954.908	p<0.001	2.261	0.133
C22:0	0.846	0.358	1118.00	p<0.001	2.175	0.141
C23:0	1.968	0.161	584.009	p<0.001	3.518	0.995
C24:0	0.006	0.937	212.453	p<0.001	1.754	0.186
C14:1	0.119	0.730	206.832	p<0.001	0.340	0.560
C15:1	2.000	0.158	7.110	0.008	2.215	0.137
C16:1	0.106	0.744	30.373	p<0.001	0.060	0.807
C17:1	8.342	0.004	1258.533	p<0.001	0.804	0.371
C20:1 n9	5.715	0.017	470.139	p<0.001	0.438	0.509
C22:1 n9	3.294	0.071	851.795	p<0.001	1.596	0.208
C18:2 n6t	1.367	0.243	15.035	p<0.001	0.023	0.879
C18:3 n3	6.864	0.009	1517.156	p<0.001	2.626	0.106
C18:3 n6	4.335	0.038	2635.954	p<0.001	5.386	0.021
C20:2	2.552	0.111	915.134	p<0.001	4.231	0.995
C20:4 n6	4.971	0.026	1392.105	p<0.001	0.318	0.573
C20:5 n3	4.770	0.029	676.08	p<0.001	0.505	0.478
C22:2	1.300	0.255	1329.27	p<0.001	2.946	0.087
